# Determining Optimal Dosage of High-Modulus Asphalt Binders Through Comprehensive Rheological Assessment Across Full Temperature Range

**DOI:** 10.3390/ma19061155

**Published:** 2026-03-16

**Authors:** Yijun Wang, Bolan Ye, Qisheng Wang, Qifeng Bai, Jiwang Jiang

**Affiliations:** 1 Henan Jiaotou Engineering Management Consulting Co., Ltd., Zhengzhou 450006, China; 13949239639@139.com; 2 School of Transportation, Southeast University, Nanjing 211189, China; 3 Henan Jiaotou Shensui Expressway Co., Ltd., Zhengzhou 450016, China; 4 Nanjing Runcheng Transportation Science Research Institute Co., Ltd., Nanjing 210008, China

**Keywords:** high-modulus asphalt, full-temperature-range evaluation, dosage optimization, rheological assessment

## Abstract

High-modulus asphalt binders are increasingly used to improve rutting resistance and enable pavement thickness reduction. Conventional binder indices do not always capture the stress-dependent response of high-modulus systems under heavy loading, and quantitative rules for selecting a high-modulus additive dosage are still limited. This study develops a full-temperature-range evaluation and dosage determination framework for high-modulus additive-modified asphalt binders (HMABs) produced on an SBS-modified base binder. Four binders were prepared with high-modulus additive dosages of 0%, 17%, 22% and 28% with a binder mass basis. High-temperature performance was evaluated by PG grading and an enhanced MSCR protocol that included 0.1, 3.2, 6.4 and 12.8 kPa. MSCR temperatures were selected based on PG results. Intermediate-temperature performance was evaluated using LAS at 25 °C with VECD-based fatigue analysis on RTFO + PAV-aged binders. Low-temperature cracking was evaluated using ABCD on PAV-aged binders at −36 °C. The results show that the high-temperature PG increased with dosage, but the 22% and 28% binders fell into the same grade, indicating limited dosage discrimination by the PG test. The enhanced MSCR test captured clearer dosage differences under higher stresses. Non-recoverable compliance decreased markedly with dosage, and stress sensitivity showed an overall decreasing trend; 6.4 kPa provided higher dosage sensitivity and lower variability than 3.2 kPa. LAS test shows a non-monotonic fatigue response in which peak shear stress and predicted fatigue life increased up to 22% and then declined at 28%. At 2.5% and 5% strain, the 22% binder increased predicted fatigue life by about 273% and 83% relative to the base binder, while at 10% strain, it was about 11% lower. ABCD results show an upward shift in critical cracking temperature and a clear reduction in fracture stress at high dosages, indicating increasing low-temperature fracture risk. Therefore, high-modulus additives markedly improve high-temperature stability but introduce full-temperature trade-offs. The proposed full-temperature-range examined framework improves performance discrimination and supports dosage selection. A target dosage of 22% is recommended, and 17~22% is suggested as an engineering-controllable range for a balanced full-temperature performance, while 28% should be treated as an upper-bound option, primarily for warm regions where rutting dominates.

## 1. Introduction

High-Modulus Asphalt Concrete (HMAC) originated from the French high-modulus asphalt system. By introducing high-viscosity binders into asphalt mixtures, this technology effectively controls permanent deformation under increasing traffic loading. It then evolved into the high-modulus mixture system represented by Enrobé à Module Élevé (EME) [1,2,3]. Since the 1980s, HMAC has been applied in pavement strengthening and maintenance projects with limited excavation depth and was later extended to base and binder courses on high-traffic roads. In some projects, it has also been used to limit asphalt layer thickness and improve rutting resistance [1,4]. The Long-Term Pavement Performance (LTPP) program in South Africa further verified its performance under extremely heavy loading and supported subsequent updates of design specifications [5]. Accordingly, HMAC has been widely adopted in Europe and other regions for heavy-duty pavements, long-life pavement structures, and structural overlays, and it is widely regarded as an effective solution for enhancing structural capacity and rutting resistance [2,5,6,7].

Currently, China boasts the world’s longest asphalt pavement network, and the development priorities have shifted from construction to maintenance, demanding enhanced durability of pavement structures [8]. However, with sustained growth in traffic demands, premature rutting and fatigue cracking have become widespread phenomena [9]. Furthermore, high-temperature and high-humidity climates exacerbate this issue, as elevated temperatures and humidity accelerate the physicochemical degradation of SBS-modified binders [10]. This directly increases the frequency and cost of maintenance and also generate greenhouse gas emissions across all phases of maintenance [11,12]. Conventional asphalt binders and common polymer-modified binders often fail to meet the performance requirements of long-life pavements, which are typical materials in highway construction [13,14]. The high-modulus target of HMAC ultimately depends on the binder system, which is high-modulus asphalt binders (HMABs). Therefore, HMABs have attracted increasing attention in China as a key material for achieving HMAC performance, and many systematic studies have been reported in this area [4,15,16].

Existing studies generally indicate three main methods to achieve a high-modulus binder system. The first is to use hard paving-grade binders to increase viscosity and stiffness. The second is to incorporate natural asphalts to enhance strength and deformation resistance. The third is to use high-modulus additives or hybrid modification strategies to enable tunable performance and engineering adaptability [1,4,17]. In China, due to limited production capacity and availability of hard binders, practice more often relies on natural asphalts or high-modulus additives to modify base binders for producing HMABs, rather than using hard binders directly [16]. Compared with direct reliance on hard binders, additive-based or hybrid methods are more practical for material sourcing, dosage control, and compatibility with different base binders. Therefore, they are more attractive in regions where hard binders are limited in supply or cost-sensitive [1,16].

Although research and engineering applications of high-modulus asphalt systems have expanded, current binder evaluation frameworks remain limited for these systems and cannot reliably capture their key performance features. Moreover, most studies focus on cross-comparisons among modifiers, while systematic and quantitative dosage optimization and transferable engineering criteria for high-modulus additives remain insufficient. Conventional DSR-based indices, such as the rutting factor (G*/sinδ) and the fatigue factor (G*sinδ), have limitations for modified binders. The rutting factor may not predict rutting response well for some modified systems. The fatigue factor is usually measured in the linear viscoelastic range at low strain and cannot directly represent damage evolution and fatigue failure under repeated loading [18,19]. The multiple stress creep and recovery (MSCR) test is considered more suitable for quantifying non-recoverable deformation and stress sensitivity. However, results from the standard stress levels of 0.1 and 3.2 kPa may still have insufficient resolution to distinguish dosage effects for high-modulus systems. Therefore, a stress-level procedure and an effective operating domain are needed for high-modulus binders [18,20,21,22]. In addition, a large amount of studies have investigated intermediate-temperature fatigue cracking using linear amplitude sweep (LAS), energy-based approaches and the Glover–Rowe parameter [18,19]. However, in the research on HMABs formed by high-modulus additives in base asphalt, the determination of additive amount often lacks the study of the additive amount gradient, which limits the development of reliable dosage–fatigue response relationships and transferable dosage criteria for engineering practice. For low-temperature evaluation, thermal cracking is a fracture failure, and it is important to select appropriate fracture-relevant indicators for cracking assessment [9]. However, BBR parameters mainly reflect stiffness and relaxation and thus provide indirect indications of cracking risk [23]. In addition, the specimen dimension and low loading levels used in the BBR test may not fully represent cracking behavior of field-scale materials at low temperatures [24].

It should be noted that the performance gain of high-modulus systems involves clear temperature-dependent trade-offs. Increasing binder stiffness typically improves resistance to permanent deformation and carrying capacity at high temperatures. It can also reduce fatigue resistance at intermediate temperatures and weaken stress relaxation and cracking toughness at low temperatures [1,7,17]. Under cold climates or large temperature gradients, very stiff systems are more prone to thermal cracking and transverse cracking. This has become a major issue to the wider application of high-modulus technology [4,7,20,25,26,27]. Therefore, HMAB design and evaluation require a full-service-temperature perspective. High-temperature stability should be improved while controlling fatigue life and low-temperature cracking risks.

## 2. Objectives and Scopes

To address the abovementioned limitations, this study adopts an SBS-modified binder as the base binder, and the specific objectives are to:

1. Establish an enhanced MSCR approach to quantify the permanent deformation resistance and stress sensitivity of high-modulus binders in high temperatures and to improve dosage discriminability within the effective operating domain.
2. Develop a comprehensive full-temperature-range evaluation framework for high-modulus modified binders to support dosage identification and engineering decision-making.

## 3. Materials and Test Methods

### 3.1. Materials and Sample Preparation

#### 3.1.1. The Base Binder

An SBS-modified asphalt binder with 3% SBS content was used as the base binder to prepare four high-modulus additive modified binders with different dosages. The technical properties of the SBS-modified base binders are summarized in Table 1.

#### 3.1.2. The High-Modulus Additive

The high-modulus additive used is primarily composed of high-molecular-weight polymers. It appears as black, spherical granules and exhibits excellent compatibility with asphalt. The physical properties of the high-modulus additive are listed in Table 2.

### 3.2. Sample Preparation

According to relevant technical guidelines and previous studies of high-modulus asphalt mixtures, the recommended additive content is typically around 1% by mass of mineral aggregate. To establish a reasonable dosage gradient around this reference value, three levels relative to aggregate masses of 0.8%, 1.0% and 1.2% were considered in this study. Because the rheological evaluation of asphalt binders is considered, these aggregate-based additive contents were converted into equivalent percentages relative to the asphalt binder mass. In addition, the total binder–aggregate ratio, including the SBS-modified binder and the high-modulus additive, was fixed at 5.2%. Therefore, the corresponding additive contents expressed as mass percentages of the asphalt binder were approximately 17%, 22%, and 28%. Meanwhile, a 0% dosage was included as a control group to enable systematic comparison of dosage effects. Accordingly, four proportions were investigated, which were 0%, 17%, 22% and 28%.

The SBS-modified asphalt binder was heated until it became fully fluid. The heating device was preheated to 170~180 °C. A high-speed homogenizing shear mixer was set to 6000~6500 rpm. The required amount of high-modulus additive was weighed according to the target content and was added into the container in several batches. The blend was sheared for 30 min and then stored in the oven for 30 min.

Short-term aging was conducted using the Rolling Thin-Film Oven (RTFO) procedure. The RTFO residue was collected and used as the short-term aged binder for subsequent tests.

### 3.3. Test Methods

#### 3.3.1. High-Temperature Performance Grade (PG) Test

A dynamic shear rheometer (DSR), Anton Paar MCR102, Shanghai, China, was employed to characterize the rheological properties of asphalt binders. A 25 mm parallel plate with a 1 mm gap was used. Both the critical temperatures of original binders and RTFO residue were tested in three groups according to AASHTO T 315 [28].

#### 3.3.2. MSCR Test

The MSCR test was used for further testing of the rheological properties using a DSR, and the temperature was selected based on the high-temperature PG results. A 25 mm sample plate with a 1 mm control height was used. The tests were performed in stress-controlled mode on RTFO-aged binders according to AASHTO T 350 [29]. Each creep–recovery cycle consisted of 1 s loading followed by 9 s unloading. To better capture the stress-dependent behavior of high-modulus binders, an enhanced MSCR protocol was adopted based on the related existing literature [30,31]. In addition to the standard stress levels of 0.1 kPa and 3.2 kPa, two higher stress levels of 6.4 kPa and 12.8 kPa were included.

#### 3.3.3. LAS Test

The LAS test was conducted adopting a DSR in strain-controlled mode. The viscoelastic continuum damage (VECD) model was applied to analyze fatigue life for different asphalts. The test consisted of two stages in accordance with AASHTO T 391 [32] The frequency sweep from 0.2 Hz to 30 Hz was first performed within the linear viscoelastic region. Subsequently, a strain sweep was applied at a constant frequency of 10 Hz. The strain amplitude increased linearly from 0.1% to 30% over a total duration of 310 s. The test temperature was 25 °C. An 8 mm sample plate with a 2 mm control height was used. The binder specimens were RTFO-aged and further aged in a pressure aging vessel (PAV) for 20 h.

The experimental results were interpreted using the viscoelastic continuum damage (VECD) framework. Damage evolution was quantified on a cycle-by-cycle basis by computing the dissipated energy from the measured shear strain, complex modulus, phase angle and loading duration. The cumulative damage intensity *D* was obtained incrementally from these parameters to capture the progressive deterioration of the material under cyclic loading.

Within this framework, the integrity parameter *C* was defined based on |G*|sinδ which serves as an indicator of the binder’s effective load-bearing capability. As damage accumulates, *C* gradually decreases, reflecting the degradation of the internal microstructure. The functional relationship between material integrity and accumulated damage was described using a power-law formulation (Equation (1)).

(1) $$C=\left|G^{*}\right|sin\delta =C_{0}-C_{1}{\left(D\right)}^{C_{2}}$$

where *C*_0_ = average value of |G*|sinδ at the 0.1% strain amplitude, and *C*_0_ = 1 when |G*|sinδ is normalized, MPa; *C*_1_ and *C*_2_ = fitting parameters of the model; |G*| = the complex shear modulus, MPa; and *δ* = the phase angle.

The material damage value at failure point *D_f_* can then be calculated using:

(2) $$D_{f}={\left(\frac{C_{0}-C\;at\;peak\;stress}{C_{1}}\right)}^{\frac{1}{C_{2}}}$$

Therefore, the fatigue life *N_f_* was calculated using:

(3) $$N_{f}=\frac{f{\left(D_{f}\right)}^{k}}{k{\left(\pi I_{D}C_{1}C_{2}\right)}^{\alpha }}{\left(\gamma_{max}\right)}^{-2\alpha }=A{\left(\gamma_{max}\right)}^{B}$$

where $k=1+(1-C_{2})\alpha$; α = rate of damage evolution; *I_D_* = |G*| at 1.0% strain amplitude, MPa; and *γ_max_* = the maximum expected strain, %.

#### 3.3.4. Asphalt Binder Cracking Device (ABCD) Test

Low-temperature cracking performance was evaluated using an asphalt binder cracking device. The test was conducted in accordance with AASHTO T 387-19 (2023) [33]. PAV-aged binders were used. The binder was heated until fully liquid and then poured into standard ring molds. The specimen was tested in a low-temperature environment of −36 °C. The sensors recorded the response to determine the ABCD cracking temperature and fracture stress of the specimen.

## 4. Results and Discussion

### 4.1. High-Temperature Rheological Performance

#### 4.1.1. High-Temperature PG Test Results

The critical temperatures and the corresponding high-temperature PGs for different contents of high-modulus additives are summarized in Table 3. As the content increases, the high-temperature PG of the SBS-modified binder increases accordingly, indicating that the additive enhances the binder’s stiffness at high temperatures. However, the binders with 22% and 28% additives exhibited the same high-temperature PG, suggesting that PG high-temperature grading cannot differentiate their high-temperature performance alone. This also indicates that the PG grading procedure has limited discriminability for high-modulus modified binders, and additional tests are required to further evaluate the high-temperature performance under different dosages.

#### 4.1.2. Effective Stress Range for the MSCR Test

**Figure 1:**
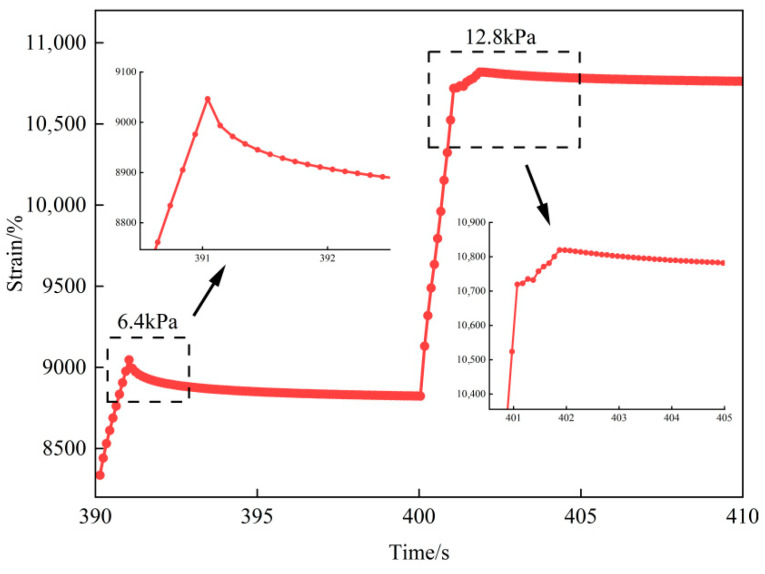
Cumulative shear strain curve at high stress levels for the binder with a 28% high-modulus dosage.

Based on the PG high-temperature grading results, the MSCR test temperatures were set to 76 °C, 82 °C, 88 °C and 94 °C. However, the indicator of the creep recovery *R* value became negative under high stress levels at certain temperatures. Figure 1 shows the strain–time curves of the last cycle at 6.4 kPa and the first cycle at 12.8 kPa for the binder with a 28% dosage at 82 °C. As the stress level increased, the material exhibited more pronounced creep deformation during the loading phase at 12.8 kPa. During unloading, the recovery strain did not decrease as expected but continued to increase. Moreover, the strain at the end of the unloading phase remained higher than the total strain reached at the end of the loading phase. As a result, the *R* value at 12.8 kPa was negative. This abnormal behavior was not observed during the unloading phase at 6.4 kPa. These results indicate that under a high temperature with high stress, the internal strain of high-modulus binders may exceed the nonlinear viscoelastic region. This can trigger the accumulation of internal shear damage, even though the material exhibits a relatively high modulus.

To accurately characterize the recoverable nonlinear viscoelastic response and the non-recoverable behavior, stress levels that produced negative *R* values were regarded as invalid. Therefore, the MSCR results obtained at those stress levels were excluded from subsequent evaluation. Accordingly, the effective stress ranges of the enhanced MSCR tests at each temperature are summarized in Table 4.

As indicated in Table 4, relatively high temperatures also contribute to the internal damage in high-modulus binders. Therefore, to comprehensively and accurately investigate the high-temperature performance of the high-modulus modified binders, the subsequent analysis of the enhanced MSCR results focuses on the tests conducted at 76 °C and 82 °C under three stress levels, which are 0.1, 3.2 and 6.4 kPa.

#### 4.1.3. Results of the Enhanced MSCR Test

**Figure 2:**
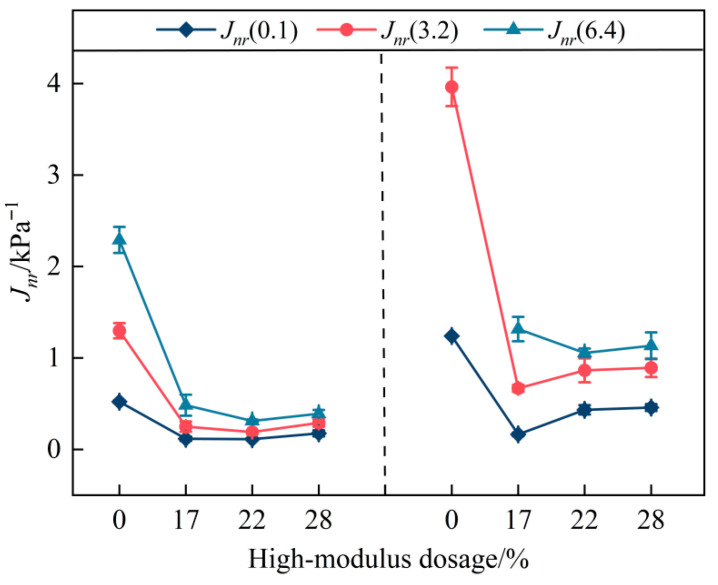
*J_nr_* results at 76 °C and 82 °C for the four binders.

The *J_nr_* value is used to quantify the deformation resistance of asphalt binders under different stress levels. A lower *J_nr_* indicates a smaller non-recoverable strain and thus a stronger resistance to permanent deformation at high temperatures. As shown in Figure 2, the *J_nr_* value increases to varying extents for all four binders as the applied stress level increases. A similar increasing trend is also observed with increasing temperature. At a given temperature, incorporating the high-modulus additive significantly reduces the *J_nr_* value of the SBS-modified binder. This indicates that the additive effectively enhances the high-temperature deformation resistance of the SBS-modified binder.

**Figure 3:**
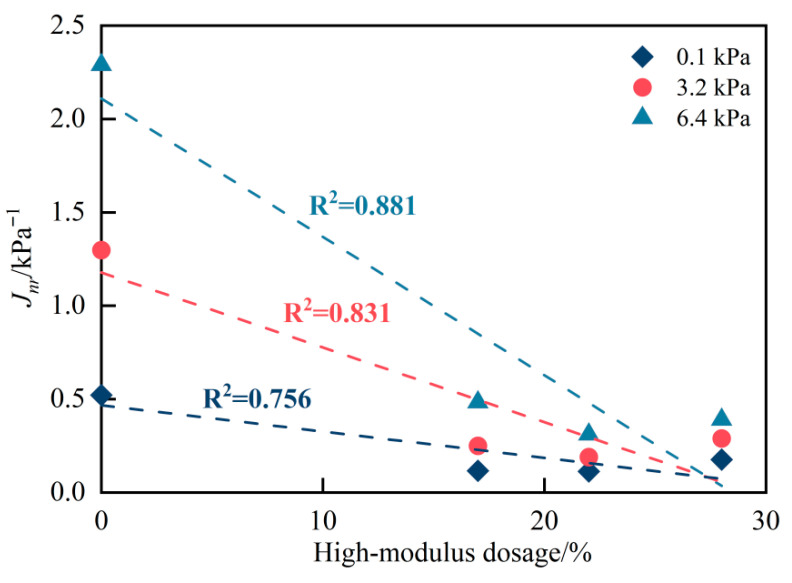
Linear regression between high-modulus dosage and *J_nr_* values at different stress levels.

In addition, as shown in Figure 3, among the three stress levels, the 6.4 kPa condition exhibits the most pronounced dosage-dependent response, as indicated by the steeper negative slope and the larger reduction range of *J_nr_* with increasing high-modulus contents. This suggests that the higher stress level is more sensitive to compositional modification.

**Figure 4:**
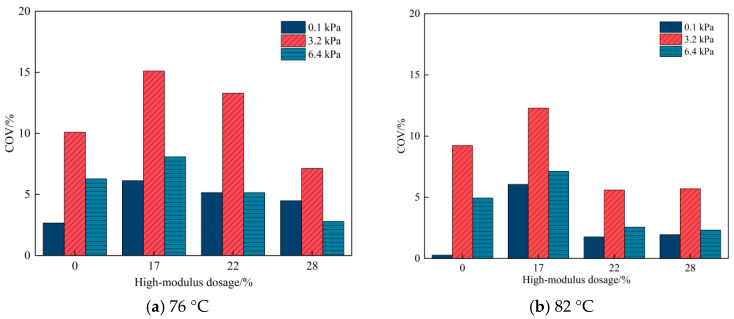
COV of *J_nr_* values across ten cycles at different stress levels for binders.

In addition, the coefficient of variation (COV) of *J_nr_* values across the ten loading cycles was calculated for each stress level. As shown in Figure 4, the *J_nr_* values at 3.2 kPa shows the highest variability, where the variability decreases markedly when the stress level increases to 6.4 kPa. This indicates that for the high-modulus binders investigated in this study, the *J_nr_* values derived from the MSCR test at 6.4 kPa are more reliable, which is similar to a previous study [34].

**Figure 5:**
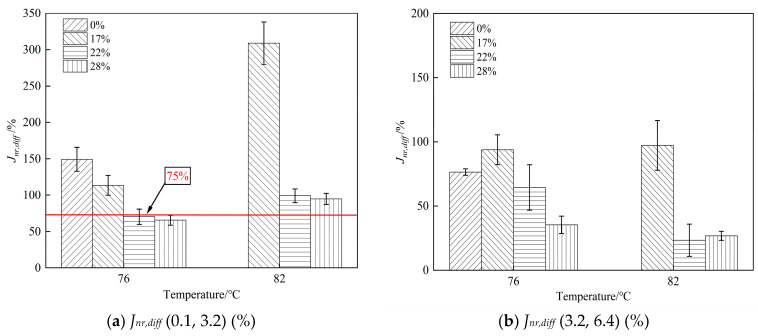
*J_nr,diff_* results for the four binders at different temperatures and stress intervals.

The parameter *J_nr,diff_* is used to quantify stress sensitivity, with larger values indicating a stronger sensitivity to changes in stress. As shown in Figure 5, the *J_nr,diff_* values of the high-modulus binders are lower than those of the SBS-modified binder in most cases, suggesting that the additive mitigates stress sensitivity. Under the 82 °C condition in the low-stress interval (0.1~3.2 kPa), the binder with a 17% dosage shows a relatively higher *J_nr,diff_*. This may suggest that the internal structure has not fully stabilized at this content level, leading to a stronger stress sensitivity response at relatively high temperatures. In addition, *J_nr,diff_* increases significantly with temperature for all four binders, indicating that stress sensitivity deteriorates at higher temperatures and that nonlinear rheological behavior becomes more pronounced. With increasing dosage, *J_nr,diff_* shows an overall decreasing trend. In particular, at 22% and 28%, *J_nr,diff_* values remain at a low level across different stress intervals, implying that a higher content contributes to improved high-temperature rheological stability.

As shown in Figure 5a, *J_nr,diff_* values at 76 °C decrease with increasing additive content. The 22% and 28% binders exhibit lower average stress sensitivity compared with the 0% and 17% binders. According to the AASHTO MP 19-10 [35] grading criterion, binders with *J_nr,diff_* values (0.1, 3.2) exceeding 75% are considered to be in a creep-damage region. When measurement variability is taken into account, the *J_nr,diff_* values of the binders with 28% dosage is below 75%, and that of the 22% approaches the 75% threshold within the experimental dispersion range, which indicates that a higher dosage can reduce the possibility of binders being in a creep-damage region. At higher temperatures, although the additive can still suppress stress sensitivity to some extent, the binders may not avoid entering the creep-damage region. Moreover, *J_nr,diff_* (3.2, 6.4) decreases further as the dosage increases, suggesting enhanced rheological stability under the higher stress range.

The recovery *R* is defined as the ratio of recoverable strain during the unloading phase to the total strain accumulated during the creep phase, reflecting the elastic recovery capability of the binder under high-temperature loading. Higher *R* values indicate stronger resistance to permanent deformation.

**Figure 6:**
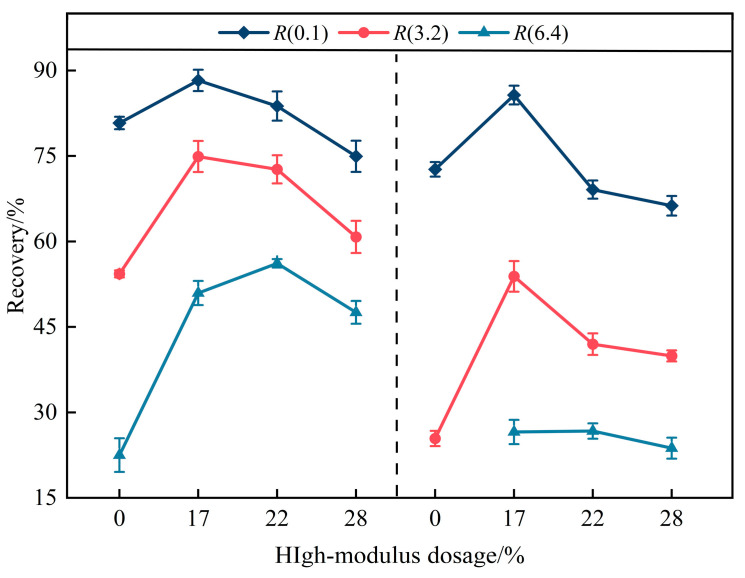
*R* results at 76 °C and 82 °C for the four binders.

As shown in Figure 6, at a given temperature, *R* decreases with increasing stress levels, indicating reduced elastic recovery under higher-loading conditions. Similarly, increasing temperature leads to a systematic reduction in recovery, reflecting enhanced viscous flow contribution. Under the extended stress level of 6.4 kPa, the 22% binder exhibits the highest mean recovery among the modified binders, suggesting improved structural stability under severe loading. However, at 82 °C, the distinction between 17% and 22% becomes less pronounced within experimental variability.

**Figure 7:**
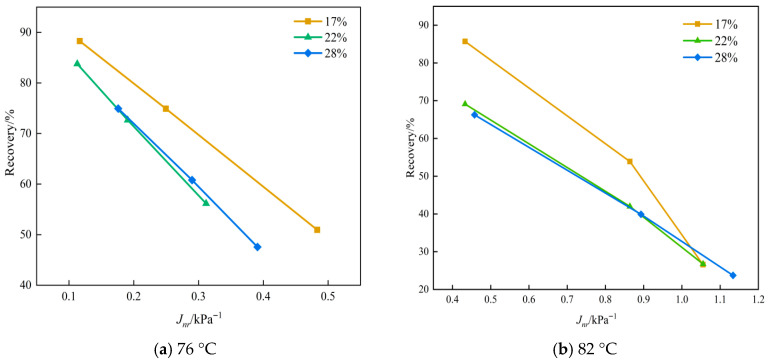
*R* versus *J_nr_* value for binders under different temperatures.

To further clarify the structural implications, the relationships between *R* and *J_nr_* are presented in Figure 7. For all modified binders, a monotonic decrease in recovery with increasing non-recoverable compliance is observed at two temperatures. This preservation of this *R*-*J_nr_* trend suggests that the high-modulus additive does not fundamentally disrupt the SBS elastic network.

To conclude, by incorporating both recovery behavior and non-recoverable compliance analysis, the MSCR evaluation is strengthened and provides a more complete assessment of high-temperature performance.

### 4.2. Intermediate-Temperature Performance

#### LAS Test Results

**Figure 8:**
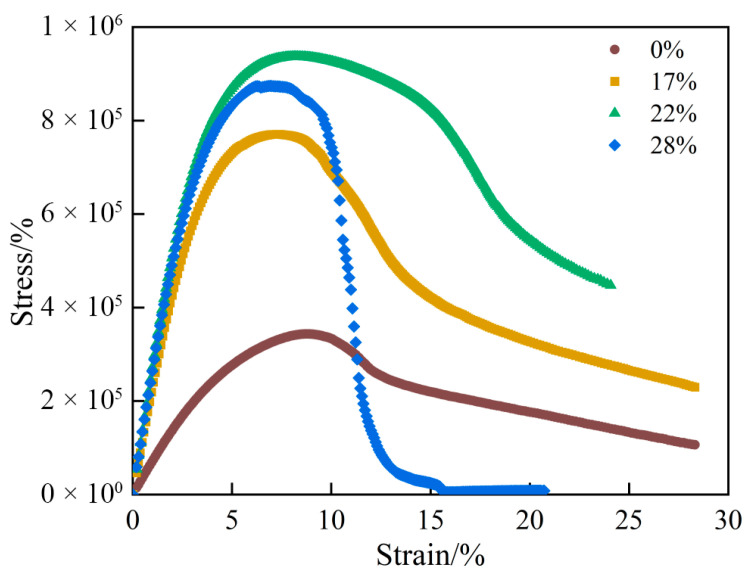
Stress–strain curves of high-modulus-modified binders.

Figure 8 presents the stress–strain curve of the LAS results. The results imply that the high-modulus additive can enhance intermediate-temperature deformation resistance to a certain extent. The differences among dosages are mainly reflected in the peak stress capacity and the post-peak response. At a content of 22%, the yield stress reaches the highest level among all binders, the post-peak reduction is markedly smaller than that of the other binders, and the corresponding stress–strain curve exhibits a broader peak. This suggests that the 22% high-modulus-modified binder sustains a relatively high stress state for a longer duration and shows better fatigue resistance. In contrast, although the 28% content also reaches a relatively high peak stress, the stress decays rapidly after the peak, indicating a higher sensitivity to fatigue damage.

LAS combined with a VECD framework is commonly used to characterize fatigue behavior by linking material integrity to damage evolution and predicting fatigue life [36]. By calculating the fitting quality between the integrity and the damage, the R^2^ values of all adhesives ranged from 0.952 to 0.995, indicating a high degree of consistency between the experimental data and the fitted VECD model. Table 5 summarizes the parameters of the VECD model.

Previous studies have suggested that lower values of the fitting parameters C_1_ and *C*_2_ are generally associated with improved fatigue resistance of asphalt binders [37]. However, it should be noted that the differences in *C*_1_ and *C*_2_ among the investigated dosages are relatively limited in this study. Therefore, relying solely on these two fitting parameters is insufficient for robustly differentiating fatigue performance. To provide a more reliable comparison, the relationship between the material integrity parameter *C* and the cumulative damage intensity *D* was further examined to characterize and compare damage evolution patterns across different contents.

**Figure 9:**
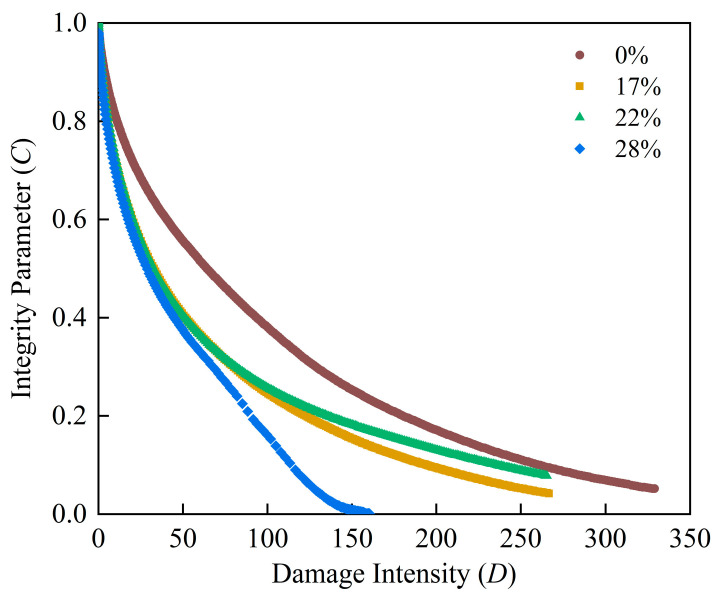
Damage curves of four binders.

Generally, for a given value of C, a larger D indicates a stronger resistance to integrity loss. As seen from Figure 9, incorporating the high-modulus additive tends to reduce the binder’s resistance to integrity loss to some extent. At low damage levels (small *D*), the curves for the 17%, 22% and 28% contents partially overlap. This implies that under minor damage, the effects of different contents are comparable. The damage curves for the 17% and 22% dosage binders decay more gradually and extend to higher damage intensity levels. Notably, the binder with 22% dosage maintains relatively higher *C* values over most damage stages. In comparison, significant integrity degradation is observed at a moderate damage level of the 28% dosage binder.

**Figure 10:**
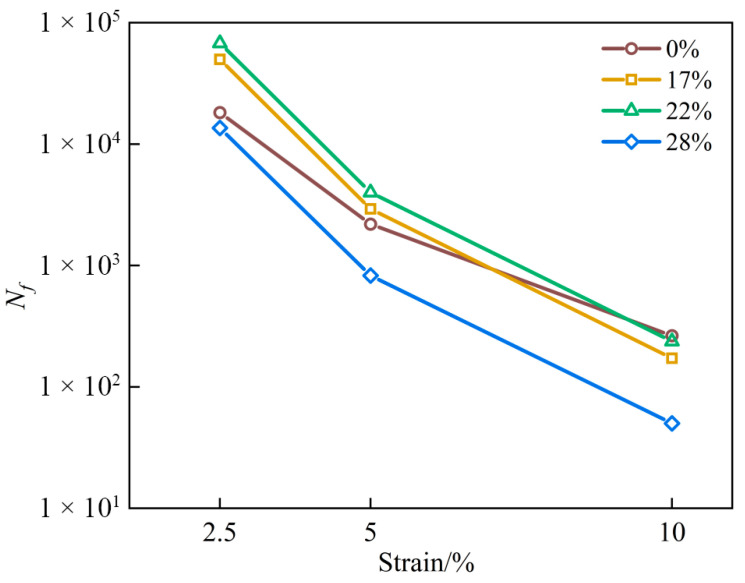
Fatigue life prediction results.

Based on the fitted parameters, the fatigue life *N_f_* is predicted using Equation (3). Figure 10 shows the results of the fatigue life analysis. The results indicate a clear increase-then-decrease trend with dosage at strain levels of 2.5, 5 and 10%. At 2.5% strain, the 22% dosage provides the longest fatigue life, corresponding to an increase of approximately 273% relative to the 0% binder. At 5% strain, the improvement remains substantial at about 83%. By 10% strain, however, the fatigue life of the 22% binder becomes about 11% lower than that of the 0% binder, which is consistent with the strain-sensitivity parameter, *B* (Table 5). The 0% binder has a smaller *B* and therefore exhibits a slower decay of *N_f_* as the strain increases. Overall, the 22% dosage delivers the most balanced and stable fatigue performance under 2.5~5% strain levels. However, excessive dosage leads to consistently reduced *N_f_* across the strain range.

### 4.3. Low-Temperature Performance

In existing studies, low-temperature performance is commonly evaluated using the BBR creep stiffness, S, and the creep slope m-value, which reflect the binder’s stiffness level and stress relaxation capability. However, low-temperature cracking ultimately manifests as fracture failure. Therefore, relying solely on the S and m-value may lead to unstable judgments or inconsistent conclusions [38]. In contrast, the ABCD test directly measures the critical cracking temperature and fracture stress under constrained cooling conditions. It characterizes low-temperature cracking risk from a fracture perspective and is more consistent with the fundamental failure mechanism. Accordingly, ABCD was employed in this study to test and evaluate the low-temperature cracking performance of high-modulus binders with different dosages.

**Figure 11:**
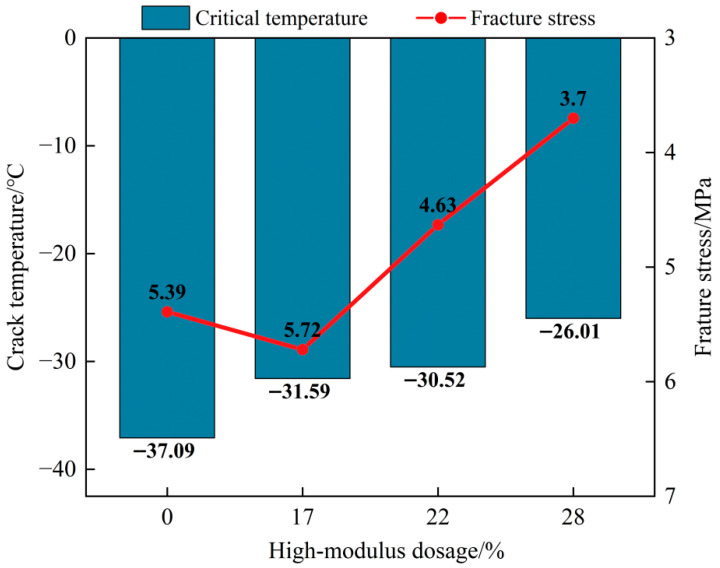
ABCD test results of critical cracking temperature and fracture stress.

As shown in Figure 11, the critical cracking temperature shifts upward overall as the dosage increases, indicating that fracture failure can be triggered at a higher temperature. Meanwhile, fracture stress demonstrates a non-monotonic dependence on dosage. A slight increase in fracture stress is observed around the 17% dosage. However, as the dosage increases further, the fracture stress decreases markedly. This suggests that a moderate dosage may enhance the fracture carrying capacity under constrained cooling, while an excessive dosage may intensify stiffening and weaken low-temperature fracture resistance. Notably, the critical cracking temperature and fracture stress do not improve simultaneously across dosages, which is consistent with observations reported for highly polymer-modified systems [23]. Therefore, low-temperature cracking risk should be assessed by jointly considering both the critical cracking temperature and fracture stress, rather than relying on a single indicator.

### 4.4. High-Modulus Dosage Optimization Based on Full-Temperature-Range Performance

By jointly constraining the results from the high-temperature enhanced MSCR tests, the intermediate-temperature LAS test and the low-temperature ABCD test, a practical content recommendation for the high-modulus dosage can be proposed.

To visualize and compare the dosage effects across temperature regions, three key indicators were converted into normalized performance scores ranging from 0 to 1 using min-max scaling across the four dosages. The high-temperature score was derived from *J_nr_* at 6.4 kPa because of the higher dosage sensitivity and relatively low variability. Since a lower *J_nr_* indicates better rutting resistance, *J_nr_* values were min-max-normalized and then reversed so that higher values indicated better performance. The intermediate-temperature score was constructed from fatigue life over the strain range of 2.5~10%. A geometric mean was used to summarize *N_f_* at 2.5%, 5% and 10% strain to account for its log-scale variation. For the low-temperature regime, a higher fracture stress is better, and the cracking temperature was converted to a negative value so that higher values indicate a lower cracking temperature and thus lower risk. Both terms were normalized by min-max scaling and combined with equal weight. It should be noted that the equal weighting scheme adopted in this study is intended for material-level comparative evaluation and the objective is to identify a balanced performance window across temperature domains rather than project-specific pavement design.

**Figure 12:**
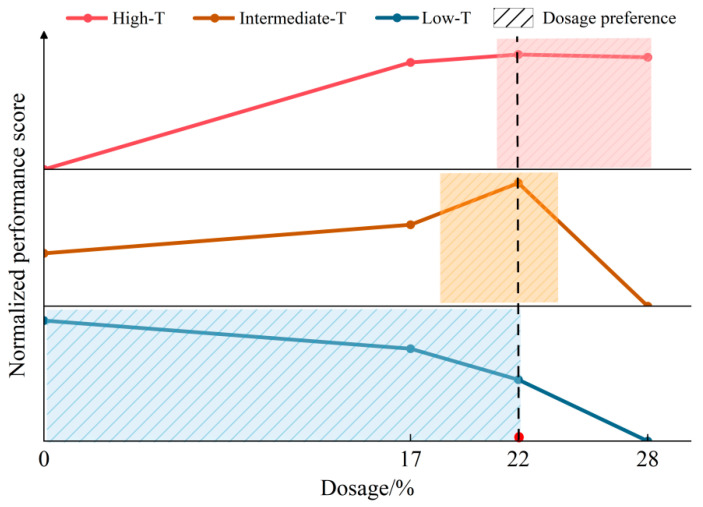
Normalized performance score and dosage preferences of three temperature regions.

Figure 12 summarizes the full-temperature dosage decision map using normalized performance scores for the high-, intermediate-, and low-temperature regimes. The dashed line marks the target dosage, which is 22%. The shaded “dosage preference” bands indicate the dosage ranges that best satisfy the dominant performance requirement in each temperature regime.

At high temperatures, the score increases sharply from 0% to 22% and then approaches a plateau, indicating that the benefit of dosage increase becomes marginal beyond 22%. The higher dosages reduce non-recoverable deformation and improve stress stability within the effective operating domain. Therefore, dosages at or above 22% are preferable for improving high-temperature performance, while 28% should be treated as an engineering upper bound. At intermediate temperatures, the score exhibits a clear non-monotonic pattern and reaches its maximum at 22%, which supports 22% as the optimal dosage. In contrast, the score drops markedly at 28%, indicating a pronounced deterioration in fatigue performance at excessive dosages. At low temperatures, the score decreases continuously with dosage, reflecting the adverse evolution of ABCD fracture indicators. This provides the primary constraint against overly high dosages and supports keeping the dosage below or around 22% when cracking risk governs.

By combining these trends, 22% is regarded as the target dosage that maximizes high-temperature stability while maintaining the best intermediate-temperature fatigue performance under the constraint of increasing the low-temperature cracking risk. In engineering practice, a dosage around of 22% is recommended to account for climatic conditions and construction variability. Moreover, the specific increase or decrease in dosage should be determined based on the project objectives and the climatic conditions of the construction site.

## 5. Conclusions

This paper addresses the issues of insufficient indicator representation, distorted service conditions and unclear methods for selecting dosages in the current evaluation system for high-modulus modified asphalt binders. It proposes a set of test stress levels for the MSCR test that are suitable for assessing high-modulus asphalt systems. Through validation with medium-temperature LAS tests and low-temperature ABCD tests, a comprehensive method for determining high-modulus agent content across the entire temperature range is established.

Across the full temperature range, a high modulus content correlates with performance response patterns. The high-temperature performance generally improves with increasing contents. The intermediate-temperature anti-fatigue performance appears as a distinct non-monotonic trend with a peak at 22% dosage. The low-temperature fracture temperature rises while the fracture stress decreases overall, indicating that high content amplifies low-temperature fracture risks. Integrating the acceptable ranges and optimal dosages across all three temperature zones, the optimal dosage for the high-temperature region is 22%, while the acceptable ranges for both intermediate and low temperatures fall within the 17~22% interval. Based on the previous analysis, this study adopts 22% as the target dosage and limits the engineering-controllable range to 17~22%. This approach maximizes comprehensive performance improvement in high-temperature deformation resistance and intermediate-temperature fatigue resistance while ensuring that low-temperature risks remain controllable. It provides an actionable basis for optimizing the content of high-modulus modified asphalt binders.

It should be noted that the conclusions in this paper are based on discrete dosage levels. Subsequent studies may build upon this foundation by conducting additional experiments with a more intensive gradients near the 22% dosage to further enhance the accuracy and robustness of the recommended content values for high-modulus additives for the SBS-modified binder system.

## Figures and Tables

**Table 1:** Main physical properties of SBS-modified binders.

Technical Indicators	Test Results	Technical Specifications
Penetration (25 °C)/0.1 mm	53.7	40~60
Softening point/°C	75.3	≥60
Ductility (5 °C)/cm	31.5	≥20
Viscosity (135 °C)/Pa·s	1.55	≤3

**Table 2:** Main physical properties of high-modulus additive.

Technical Indicators	Test Results	Technical Specifications
Apparent relative density	2.711	≥2.5
Density	1.08	1~1.1
Water content/%	0.4	≥1
Hydrophilicity coefficient/%	0.56	<1
Plasticity index	2.6	<4

**Table 3:** High-temperature PG results for the four binders.

Dosage (%)	Binder Condition	Critical Temperature (℃)	High-Temperature PG (℃)
0	Unaged	90.3	76
	RTFO	80.0	
17	Unaged	93.9	88
	RTFO	91.9	
22	Unaged	94.8	94
	RTFO	94.2	
28	Unaged	95.6	94
	RTFO	96.5	

**Table 4:** Effective stress range analysis results.

Temperature (℃)	Effective Stress Levels (kPa)			
	0%	17%	22%	28%
76	0.1/3.2/6.4	0.1/3.2/6.4	0.1/3.2	0.1
82	0.1/3.2/6.4	0.1/3.2/6.4	0.1/3.2	0.1/3.2
88	0.1/3.2/6.4	0.1/3.2/6.4	0.1/3.2/6.4	0.1/3.2
94	0.1/3.2/6.4	0.1/3.2/6.4	0.1/3.2/6.4	0.1/3.2

**Table 5:** Fitted VECD model parameters for the binders.

Dosage (%)	*C*_0_	*C*_1_	*C*_2_	*A*	*B*	*α*
0	1	0.057	0.507	2.98 × 10^5^	3.051	1.526
17	1	0.103	0.419	2.11 × 10^6^	4.089	2.044
22	1	0.100	0.421	2.85 × 10^6^	4.080	2.040
28	1	0.103	0.452	5.48 × 10^5^	4.037	2.018

## Data Availability

The raw data supporting the conclusions of this article will be made available by the authors on request.
